# Diphtheria-like Disease Caused by Toxigenic *Corynebacterium ulcerans* Strain

**DOI:** 10.3201/eid2007.140216

**Published:** 2014-07

**Authors:** Vartul Sangal, Leena Nieminen, Barbara Weinhardt, Jane Raeside, Nicholas P. Tucker, Catalina-Diana Florea, Kevin G. Pollock, Paul A. Hoskisson

**Affiliations:** Strathclyde Institute of Pharmacy and Biomedical Sciences, University of Strathclyde, Glasgow, Scotland, UK (V. Sangal, L. Nieminen, N.P. Tucker, P.A. Hoskisson);; Faculty of Health and Life Sciences, Northumbria University, Newcastle upon Tyne, UK (V. Sangal);; Health Protection Scotland, Glasgow (K.G. Pollock);; Royal Alexandra Hospital, Paisley, UK (B. Weinhardt, J. Raeside, C.-D. Florea)

**To the Editor:** Toxigenic *Corynebacterium ulcerans* is an increasingly reported cause of diphtheria in the United Kingdom and is often associated with a zoonotic origin (*1*,*2*). Here, we report a case of diphtheria caused by toxigenic *C. ulcerans* in a woman, 51 years of age, from Scotland, UK, who was admitted to a hospital in August 2013 with a swollen, sore throat and a gray-white membrane over the pharyngeal surface. The patient had returned from a 2-week family holiday in the state of Florida, United States, before the admission and also reported recent treatment of a pet dog for pharyngitis. The patient was believed to have been vaccinated against diphtheria during childhood. She was immediately admitted to an isolation ward and treated with a combination of clindamycin, penicillin, and metronidazole. 

Microscopic examination of the throat biofilm (collected by using a swab) showed gram-positive bacilli; swab samples from the exudative membrane and throat produced small, black colonies indicative of *Corynebacterium* spp. on Hoyle medium. Further efforts to identify the strain by using VITEK MS and VITEK2 ANC card systems (bioMérieux, Marcy l’Etoile, France) to evaluate the swab samples suggested that the infection was caused by either *C. ulcerans* or *C. pseudotuberculosis* (50% CI). The isolate detected from this process was sent to the *Streptococcus* and Diphtheria Reference Unit, Public Health England, Colindale, UK, and was confirmed to be a toxigenic *C. ulcerans* strain that we designated RAH1. Throat swab samples were collected from family members of the patient and were negative for *C. ulcerans*. The family dog was not tested for presence of the organism, although it is known that *C. ulcerans* infections are often of a zoonotic nature (*1*,*2*). After treatment, the patient made a full recovery.

Toxigenic *C. ulcerans* can produce both diphtheria-like and Shiga-like toxins (*3*); to identify the genetic basis of toxin production and other potential virulence factors in this strain, a whole genome sequencing approach was applied to the isolate. The genome was sequenced by using an Ion PGM System (Thermo Fischer Scientific, Loughborough, Leicestershire, UK) and resulting reads (2,965,044 reads, ≈90x coverage. Data are available on GenBank SRA: high-throughput DNA and RNA sequence read archive (http://www.ncbi.nlm.nih.gov/Traces/sra/sra.cgi?view=search_obj, accession no.: SRR1145126) and were mapped onto the published genome sequences of a Shiga-like toxin–producing clinical isolate 809, asymptomatic canine strain BR-AD22 (*3*), and diphtheria-like toxin–producing strain 0102 (*4*). Most of the previously identified virulence genes (*3*,*4*) were present in the patient isolate (Table). The *tox* gene, encoding diphtheria toxin, was present, which verified the diphtheria-like disease in the patient. The *rbp* gene, responsible for Shiga toxin–like ribosome-binding protein, was absent. However, strain RAH1 also possessed the venom serine protease gene (*vsp2*), which, in *C. ulcerans* strain 809, has been implicated in the increased virulence in humans. The *tox* gene was present in a prophage that showed similarities to ΦCULC809I (*3*) and ΦCULC0102-I (*4*). Genome-based phylogenetic analysis of the RAH1 strain (ClonalFrame analysis [*5*]) and strains 809, BR-AD22, and 0102 indicates a much wider phylogenetic diversity of *C. ulcerans* strains than previously appreciated (data not shown).

**Table:** Virulence genes associated with *Corynebacterium ulcerans* present in strain RAH1 isolated from patient with diphtheria-like disease, 2013, United Kingdom*

Gene	Strains	Strain RAH1	Potential function
*tox*	0102	P	Diphtheria-like toxin
*rbp*	809	A	Shiga toxin–like ribosome binding protein
*cpp*	809, BR-AD22, 0102	P	Corynebacterial protease CP40, protective antigen against caseous lymphadenitis
*pld*	809, BR-AD22, 0102	P	Toxic phospholipase D
*spaF*	809, BR-AD22, 0102	P	Surface-anchored protein, pilus tip protein
*spaE*	809, BR-AD22, 0102	P	Surface-anchored protein, minor pilin subunit
*spaD*	809, BR-AD22, 0102	P	Surface-anchored protein, major pilin subunit
*spaC*	809, BR-AD22, 0102	P†	Surface-anchored protein, pilus tip protein
*spaB*	809, BR-AD22, 0102	P	Surface-anchored protein, minor pilin subunit
*rpfI*	809, BR-AD22, 0102	P	Resuscitation-promoting factor interacting protein
*cwlH*	809, BR-AD22, 0102	P	Cell wall–associated hydrolase
*nanH*	809, BR-AD22, 0102	P	Neuraminidase, glycosyl hydrolases
*vsp1*	809, BR-AD22	P	Venom serine protease
*vsp2*	809	P	Venom serine protease
*tspA*	809, BR-AD22	P	Trypsin-like serine protease

*P, present; A, absent. †≈700 bp deletion.

This case raises the issue of waning vaccine protection in older patients and suggests that toxin-mediated corynebacterial disease remains a threat to public health. The declining costs of next-generation sequencing and availability of easy-to-handle bioinformatics tools emphasize the suitability of deep-sequencing technology for rapid diagnostics and for the development of high-resolution genotyping. It is time for the wider introduction of this technology into public health investigations.
